# Gene expression profile in obesity and type 2 diabetes mellitus

**DOI:** 10.1186/1476-511X-6-35

**Published:** 2007-12-14

**Authors:** Undurti N Das, Allam A Rao

**Affiliations:** 1 UND Life Sciences, 13800 Fairhill Road, #321, Shaker Heights, OH 44120, USA; 2 Department of Computer Science and Systems Engineering, Andhra University, Visakhapatnam-530003, India

## Abstract

Obesity is an important component of metabolic syndrome X and predisposes to the development of type 2 diabetes mellitus. The incidence of obesity, type 2 diabetes mellitus and metabolic syndrome X is increasing, and the cause(s) for this increasing incidence is not clear. Although genetics could play an important role in the higher prevalence of these diseases, it is not clear how genetic factors interact with environmental and dietary factors to increase their incidence. We performed gene expression profile in subjects with obesity and type 2 diabetes mellitus with and without family history of these diseases. It was noted that genes involved in carbohydrate, lipid and amino acid metabolism pathways, glycan of biosynthesis, metabolism of cofactors and vitamin pathways, ubiquitin mediated proteolysis, signal transduction pathways, neuroactive ligand-receptor interaction, nervous system pathways, neurodegenerative disorders pathways are upregulated in obesity compared to healthy subjects. In contrast genes involved in cell adhesion molecules, cytokine-cytokine receptor interaction, insulin signaling and immune system pathways are downregulated in obese. Genes involved in signal transduction, regulation of actin cytoskeleton, antigen processing and presentation, complement and coagulation cascades, axon guidance and neurodegenerative disorders pathways are upregulated in subjects with type 2 diabetes with family history of diabetes compared to those who are diabetic but with no family history. Genes involved in oxidative phosphorylation, immune, nervous system, and metabolic disorders pathways are upregulated in those with diabetes with family history of diabetes compared to those with diabetes but with no family history. In contrast, genes involved in lipid and amino acid pathways, ubiquitin mediated proteolysis, signal transduction, insulin signaling and PPAR signaling pathways are downregulated in subjects with diabetes with family history of diabetes. It was noted that genes involved in inflammatory pathway are differentially expressed both in obesity and type 2 diabetes. These results suggest that genes concerned with carbohydrate, lipid and amino acid metabolic pathways, neuronal function and inflammation play a significant role in the pathobiology of obesity and type 2 diabetes.

## Introduction

Metabolic syndrome X is characterized by (a) abdominal obesity, (b) atherogenic dyslipidemia, (c) raised blood pressure, (d) insulin resistance with or without glucose intolerance, (e) pro-inflammatory state, and (f) prothrombin state. Thus, subjects who have abdominal obesity, atheroslcerosis, insulin resistance and hyperinsulinemia, hyperlipidemias, endothelial dysfunction, essential hypertension, type 2 diabetes mellitus, and coronary heart disease (CHD) are considered to have metabolic syndrome X. Other features of metabolic syndrome X also include: hyperfibrinogenemia, increased plasminogen activator inhibitor-1 (PAI-1), low tissue plasminogen activator, nephropathy, micro-albuminuria, and hyperuricemia. The incidence of metabolic syndrome X is increasing, and the cause(s) for this increasing incidence is not clear. Although genetics could play an important role in the higher prevalence of metabolic syndrome X, it is not clear how genetic factors interact with environmental and dietary factors to increase its incidence. Since obesity and type 2 diabetes mellitus occur together and can run in families, it will be interesting to study gene expression profiles in subjects who have obesity and type 2 diabetes mellitus with and without family history to know the role of genetics in the pathobiology of these two diseases.

Obesity is a low-grade systemic inflammatory condition [1,2]. Plasma levels of C-reactive protein (CRP), tumor necrosis factor-α (TNF-α), and interleukin-6 (IL-6), markers of inflammation are elevated in subjects with obesity, insulin resistance, essential hypertension, type 2 diabetes, and CHD both before and after the onset of these diseases [3-9]. Overweight children and adults showed an increase in CRP concentration compared with normal weight children [3]. In these subjects, a direct correlation between the degree of adiposity and plasma CRP levels was noted. Elevated CRP concentrations has been shown to be associated with increased risk for CHD, ischemic stroke, peripheral arterial disease, and ischemic heart disease mortality in healthy men and women. A strong relation between elevated CRP levels and cardiovascular risk factors: fibrinogen, and HDL cholesterol was also reported. Furthermore, weight reduction and/or exercise decrease serum concentrations of TNF-α and simultaneously a reduction in the risk of CHD is also noted. This is supported by the observation that a negative correlation exists between plasma TNF-α and HDL cholesterol, glycosylated hemoglobin, and serum insulin concentrations that could explain as to why CHD is more frequent in obese compared to healthy or lean subjects [3,9]. Despite these evidences showing the relationship between inflammatory markers and obesity, type 2 diabetes mellitus, and CHD, the exact genetic relationship between these diseases is not clear. Hence in the present study, we performed gene expression profile in subjects with obesity and type diabetes mellitus with and without family history.

## Materials and methods

Blood samples were obtained from 6 subjects. Of which: one was a healthy normal (H), one was healthy but was overweight (HO), one was obese (O), two were having type diabetes with no family history of diabetes (DNPH: DNPH1 and DNPH2), and one had type 2 diabetes whose parents were also diabetic (DPH). All these subjects were matched for age, gender, and body mass index. RNA was extracted from the peripheral blood leukocytes from these subjects, hybridized on Human 40K OciChip array (Ocimum Biosolutions, Hyderabad, India). Gene expression values were obtained after quantification of TIFF images. Data has 40,320 × 6 data points. Empty spots and control probes were removed before proceeding with data analysis. This study was approved by the Ethics Committee of Andhra University, Visakhapatnam, India., and the consent of all the participants was obtained.

### Analysis performed

The data obtained was analyzed by two methods: (a) Differential expression analysis, and (b) Functional classification of differentially expressed genes.

### Differential expression analysis

The primary objective of the study of gene expression profiles using microarray is to assess the mRNA transcript levels of samples under different experimental conditions and detect significant difference in expression levels of various genes across samples. When the number of replicates for each condition is adequate, the identification of differentially expressed genes is meaningful. However, in majority of instances, there are no or limited replicates due to practical constraints of cost and feasibility. In such an instance, appropriate statistical techniques are performed to arrive at information on differentially expressed genes.

For experiments with single sample in different conditions, it is assumed that the log intensity values of gene expression for the two samples are linearly related, following bivariate normal distribution, contaminated with outliers. In a contaminated bivariate distribution, the main body of the data is characterized by bivariate normal distribution and constitutes regular observations. The non-regular observations, described as outliers, represent systematic deviations. These outliers are often suspected as possible candidates for differential expression genes [10,11]. We have used this approach consisting of two-stages to detect outliers from bivariate population and determining differentially expressed candidate genes from these outliers. This approach provides the fold-change value considering the scatter of observations and thereby provides up and down regulated genes across the samples.

### Methodology of analysis of data

#### Multivariate outlier detection

Outlier detection is one of the important tasks in any data analysis, which describes abnormalities in the data. Many methods have been proposed in the literature for detecting univariate outliers based on robust estimation of location and scale parameters [12-14]. The standard method for multivariate outlier detection involves robust estimation of parameters in the Mahalanobis distance (MD) measure and then comparing MD with the critical value of X^2 ^distribution. The values larger than the critical value are treated as outliers of the distribution.

#### Mahalanobis distance

The shape and the size of multivariate data are quantified by the covariance matrix, which is taken into account in the Mahalanobis distance. Thus, for a multivariate sample X_ij_, where *i *= 1, 2, 3,... n (number of genes) and *j *= 1, 2, 3... p (number of samples), the Mahalanobis distance is defined as,

MD_1 _= ((x_ij_-m)^T^C^-1^(x_ij_-m))^0.5^

where *m *is the estimated multivariate location parameter and C is the estimated covariance matrix. For multivariate normal data, the squared MD values are approximately chi-square distributed with *p *degrees of freedom. Multivariate outliers can now be defined as the observations having large (squared) MD values. A quantile for a chi-square distribution can be fixed (say 95%) and the observations with MD values greater than the chi-square cut-off at 95% are considered as outliers. The location and the covariance parameters are estimated using robust estimation methods. One of the well-known methods of estimation viz. Minimum Covariance Determinant (MCD) has been used in the study.

#### Minimum covariance determinant (MCD)

The MCD estimator is determined by that subset of observations of size *h*, which minimizes the determinant of the covariance matrix computed only from the *h *observations. The location estimator is the average of these *h *observations, whereas the scatter estimate is proportional to the variance covariance matrix. As a compromise between robustness and efficiency, usually h = 0.75n (*n *is the sample size) is used in the analysis. The distances obtained after using robust estimators are referred as robust distances (RD). Rousseeuw and Van Zomeren [15] have used these RDs for multivariate outlier detection, such that if the squared RD for an observation is larger than the cut-off, say χ^2^_p;97.5%_' it can be declared as an outlier.

The location and scale parameters of the scatter can be obtained using MCD and accordingly the robust distance (RD) for each observation (gene) could be obtained using equation (1). An empirical distribution function (EDF) is obtained for RD, which is compared with the chi-square distribution function for two degrees of freedom. For multivariate normally distributed data, the empirical distribution converges to the theoretical one. Hence, the tails of the distribution are often compared for detecting outliers. The tails will be defined by small δ = χ^2^_p;1-α _for a certain small α (say 0.05). The vertical line in the figure indicates the cut-off value of chi-square for two degrees of freedom for 95% quantile. The observations with RD values greater than the cut-off are declared as outliers. Upon identifying the bivariate outliers, the next task is to identify those outlier genes that are differentially expressed across the two samples. The stage II level analysis deals with identifying a set of outlying genes that are differentially expressed.

#### Stage II: Univariate outliers detection

The univariate outliers detection analysis is performed as follows:

Let S denote the original set of observations. Let S_out _and S_in _be the subsets of S containing outlier and inlier observations respectively. Thus,

S_out _∪ S_in _= S and S_out _∩ S_in _= {∅}, i.e. the two subsets are mutually exclusive.

We denote:

S_out _= {(log_2_(X_i1_), log_2_(X_i2_))/MD_i _> c for I = 1, 2, 3..... n}

and

S_in _= {(log_2_(X_i1_), log_2_(X_i2))_/MD_i _ϕ c for I = 1, 2, 3..... n}

where 'c' is the cut-off for a given quantile and n is the total number of genes.

We define a statistic,

Z=log⁡2(X2X1)=log⁡2(X2)−log⁡2(X1)
 MathType@MTEF@5@5@+=feaafiart1ev1aaatCvAUfKttLearuWrP9MDH5MBPbIqV92AaeXatLxBI9gBaebbnrfifHhDYfgasaacPC6xNi=xI8qiVKYPFjYdHaVhbbf9v8qqaqFr0xc9vqFj0dXdbba91qpepeI8k8fiI+fsY=rqGqVepae9pg0db9vqaiVgFr0xfr=xfr=xc9adbaqaaeGacaGaaiaabeqaaeqabiWaaaGcbaGaeeOwaOLaeyypa0JagiiBaWMaei4Ba8Maei4zaC2aaSbaaSqaaiabikdaYaqabaGcdaqadaqcfayaamaalaaabaGaeeiwaG1aaSbaaeaacqaIYaGmaeqaaaqaaiabbIfaynaaBaaabaGaeGymaedabeaaaaaakiaawIcacaGLPaaacqGH9aqpcyGGSbaBcqGGVbWBcqGGNbWzdaWgaaWcbaGaeGOmaidabeaakiabcIcaOiabbIfaynaaBaaaleaacqaIYaGmaeqaaOGaeiykaKIaeyOeI0IagiiBaWMaei4Ba8Maei4zaC2aaSbaaSqaaiabikdaYaqabaGccqGGOaakcqqGybawdaWgaaWcbaGaeGymaedabeaakiabcMcaPaaa@4F0A@

which is the log of the ratio of intensity values for different genes for the two samples. Here X_1 _is treated as reference, while X_2 _is treated as test sample. The statistic provides a measure of differential expression (DE) of genes across the samples. The genes showing at least k-fold change (usually k = 2, i.e. Z = 1) across the samples are considered to be DE genes. The appropriate choice of k is important since it influences the number of DE genes. Here we propose a rationale for selecting *k *for a given percentage of bivariate outliers.

We generate values for the statistic for the entire set as,

Z={zi;i=1,2,3,........n}={log⁡2(Xi2/Xi1);i=1,2,3,.....n}
 MathType@MTEF@5@5@+=feaafiart1ev1aaatCvAUfKttLearuWrP9MDH5MBPbIqV92AaeXatLxBI9gBaebbnrfifHhDYfgasaacPC6xNi=xI8qiVKYPFjYdHaVhbbf9v8qqaqFr0xc9vqFj0dXdbba91qpepeI8k8fiI+fsY=rqGqVepae9pg0db9vqaiVgFr0xfr=xfr=xc9adbaqaaeGacaGaaiaabeqaaeqabiWaaaGcbaqbaeWabiqaaaqaaiabbQfaAjabg2da9iabcUha7jabbQha6naaBaaaleaacqqGPbqAaeqaaOGaei4oaSdcbiGae8xAaKMaeyypa0JaeGymaeJaeiilaWIaeGOmaiJaeiilaWIaeG4mamJaeiilaWIaeiOla4IaeiOla4IaeiOla4IaeiOla4IaeiOla4IaeiOla4IaeiOla4IaeiOla4Iae8NBa4MaeiyFa0habaGaeyypa0Jaei4EaSNagiiBaWMaei4Ba8Maei4zaC2aaSbaaSqaaiabikdaYaqabaGccqGGOaakcqqGybawdaWgaaWcbaGaeeyAaKMaeGOmaidabeaakiabc+caViabbIfaynaaBaaaleaacqqGPbqAcqaIXaqmaeqaaOGaeiykaKIaei4oaSJaemyAaKMaeyypa0JaeGymaeJaeiilaWIaeGOmaiJaeiilaWIaeG4mamJaeiilaWIaeiOla4IaeiOla4IaeiOla4IaeiOla4IaeiOla4IaeeOBa4MaeiyFa0haaaaa@67D6@

For a perfect linear relationship between the two samples, the Z statistic becomes residual following normal distribution with mean *m *and variance S_e_^2'^.

The statistic is used to obtain Mahalanobis distance measure as,

MDi∗=(Zi−mSe)2 for i=1,2,3.......n
 MathType@MTEF@5@5@+=feaafiart1ev1aaatCvAUfKttLearuWrP9MDH5MBPbIqV92AaeXatLxBI9gBaebbnrfifHhDYfgasaacPC6xNi=xI8qiVKYPFjYdHaVhbbf9v8qqaqFr0xc9vqFj0dXdbba91qpepeI8k8fiI+fsY=rqGqVepae9pg0db9vqaiVgFr0xfr=xfr=xc9adbaqaaeGacaGaaiaabeqaaeqabiWaaaGcbaGaemyta0Kaemiraq0aa0baaSqaaiabdMgaPbqaaiabgEHiQaaakiabg2da9maabmaajuaGbaWaaSaaaeaacqWGAbGwdaWgaaqaaiabdMgaPbqabaGaeyOeI0IaemyBa0gabaGaem4uam1aaSbaaeaacqWGLbqzaeqaaaaaaOGaayjkaiaawMcaamaaCaaaleqabaGaeGOmaidaaOGaeeiiaaIaeeOzayMaee4Ba8MaeeOCaiNaeeiiaaIaeeyAaKMaeyypa0JaeGymaeJaeiilaWIaeGOmaiJaeiilaWIaeG4mamJaeiOla4IaeiOla4IaeiOla4IaeiOla4IaeiOla4IaeiOla4IaeiOla4IaeeOBa4gaaa@5127@

The transformed distance measure is supposed to follow chi-square distribution with one degree of freedom. The empirical distribution function of MD* could be obtained and compared with that of the cumulative distribution of chi-square with one degree of freedom. A cut-off could be selected for MD* such that the observations greater than the cut-off could be declared as outliers. We search for an optimal cut-off, so that the univariate subset of outliers does not include any of the bivariate inliers. In other words, if R_out _is a subset of univariate outliers and S_in _the subset of bivariate inliers of S, then the optimal cut-off could be obtained as,

C*_opt _= inf[*C*_i_*/*R*_*out *_⋂ *S*_*in *_= {∅}]

**Figure 1:**
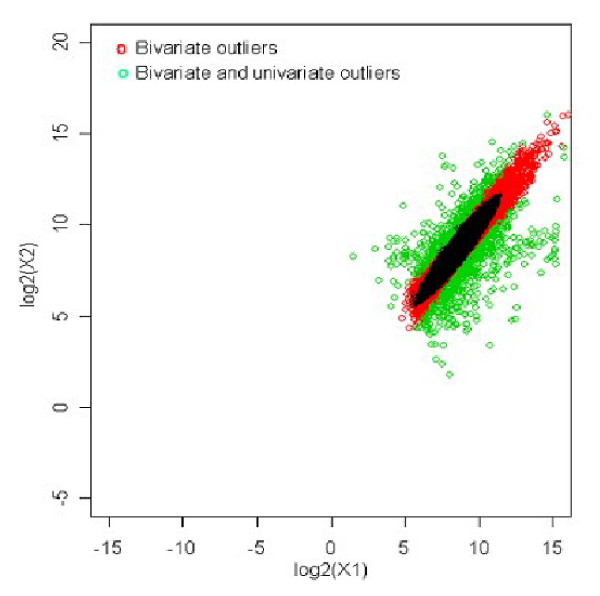
Outliers obtained using bivariate and univariate approaches.

The optimal cut-off could be obtained programmatically thereby yielding a set of univariate outliers that overlap with a subset of multivariate outliers. Figure 1 shows the common outliers obtained by both the methods for an optimal cut-off value of 6.15.

The probability value for this cut-off could be obtained by referring to chi-square distribution with one degree of freedom, which is 0.0132. Thus, 1.3% of the genes are outliers and are differentially expressed across the two samples. The observations indicated by green spots is the subset of bivariate outliers, which could have been differentially expressed across the samples. The cut-off value could be used in equation (2) to obtain the z-value as,

Z=(Se)C′opt+m
 MathType@MTEF@5@5@+=feaafiart1ev1aaatCvAUfKttLearuWrP9MDH5MBPbIqV92AaeXatLxBI9gBaebbnrfifHhDYfgasaacPC6xNi=xI8qiVKYPFjYdHaVhbbf9v8qqaqFr0xc9vqFj0dXdbba91qpepeI8k8fiI+fsY=rqGqVepae9pg0db9vqaiVgFr0xfr=xfr=xc9adbaqaaeGacaGaaiaabeqaaeqabiWaaaGcbaGaemOwaOLaeyypa0JaeiikaGIaem4uam1aaSbaaSqaaiabdwgaLbqabaGccqGGPaqkdaGcaaqaaiqbdoeadzaafaWaaSbaaSqaaiabd+gaVjabdchaWjabdsha0bqabaaabeaakiabgUcaRiabd2gaTbaa@3AB5@

**Figure 2:**
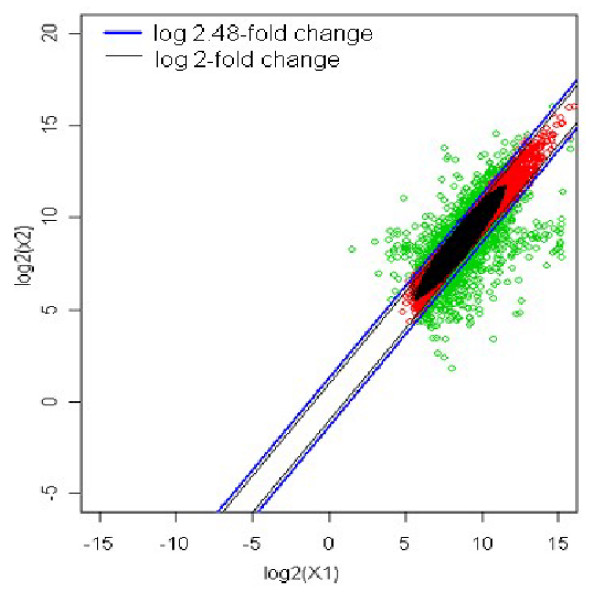
The up and down regulated genes for 2.48- and 2-log fold change thresholds.

This z-value determines the log fold change resulting into bivariate outliers that could be the potential candidates for differential expression. In the above case, z value is 1.31, suggests that the bivariate outliers with fold change of 2.5 (21.31 = 2.48) and above across the samples could be the DE candidates. Figure 2 shows thresholds for log 2.48-fold change and log 2-fold change. The log 2-fold change includes large number of bivariate inliers, which are eliminated by log 2.48-fold change. The observations above log 2.48-fold are declared as up regulated with reference to X_1_, while those below log 0.4-fold are declared as down regulated.

Thus, the approach described above identifies DE candidates amongst those observations, which are non-regular for some predefined percentile value and thereby provides a rationale for selecting the k-fold change.

#### Analysis of the data obtained in the present study

Applying these principles to the present study, in the present context, there are six individuals, one from each of the categories namely healthy (H), healthy with obesity (H&O), obesity only (O), diabetes with parental history (DPH) and two individuals having diabetes without parental history (DNPH1 and DNPH2). The expression levels of 39,400 genes for each individual were obtained and compared pair wise, resulting into fifteen combinations. The analysis was carried out for each of these combinations independently following the afore stated approach. Prior to analysis, the data for each combination was normalized using Loess normalization. Below we present the analysis for each combination along with the interpretations.

## Results

### 1. Healthy (reference) *vs*. Healthy with overweight (test sample) [H *vs *HO]

**Figure 3:**
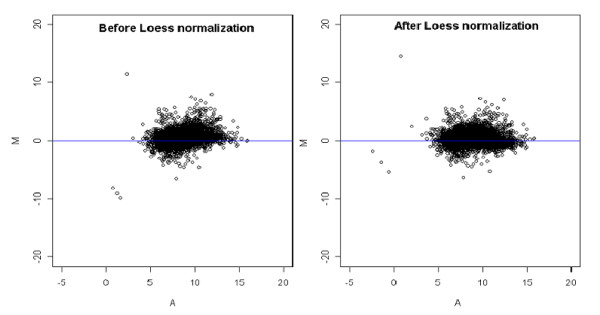
MA-plots showing scatter of expression values before and after Loess normalization for healthy *vs*. healthy with obesity comparison.

**Figure 4:**
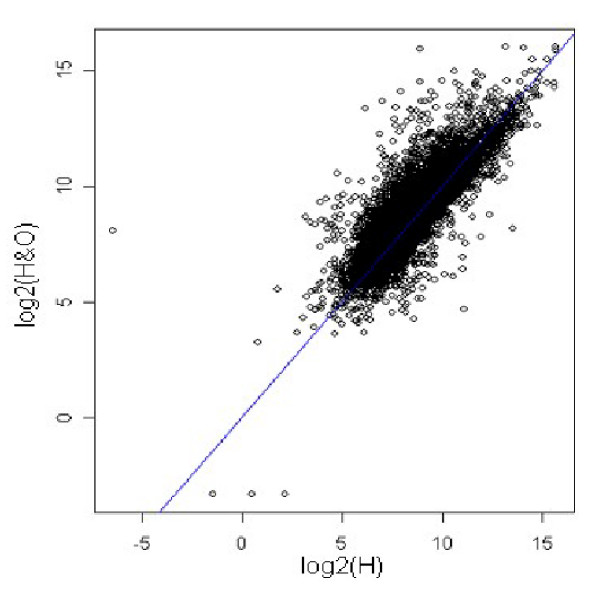
Scatter plot of log intensities for healthy *vs. *healthy with obesity comparison after Loess normalization.

**Figure 5:**
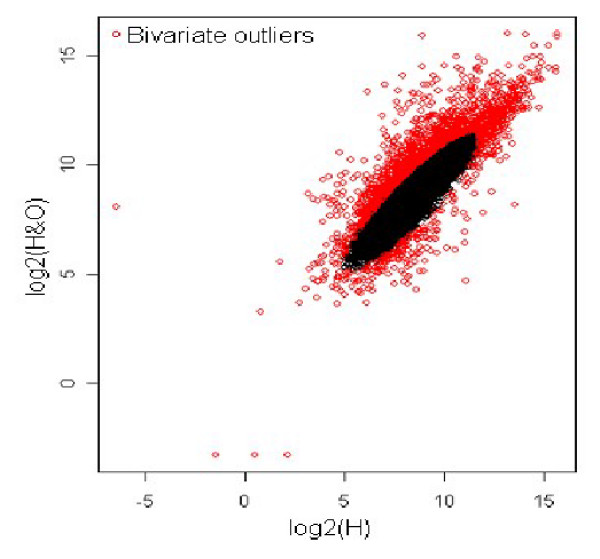
Bivariate outliers based on Mahalanobis distance measure for p = 0.10 for healthy *vs*. healthy with obesity comparison.

In the first step, the log intensity values of the gene expression for the two samples were preprocessed using loess method, in order to remove any measurement bias in the experiment. Figure 3 shows the MA-plots for before and after Loess normalization. Upon normalizing the expression values for the two samples, the scatter plot of log intensity values was obtained as shown in Figure 4. The scatter plot gives the bivariate distribution along with contaminated observations (genes)/outliers. The Mahalanobis distance measure was used to identify outliers for p = 0.10. Thus out of 39,400 genes, 3,940 genes were identified as outliers as indicated by red spots in Figure 5.

**Figure 6:**
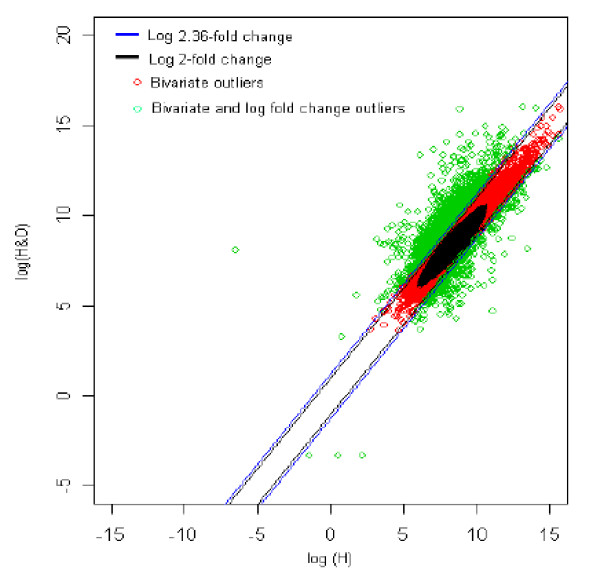
The thresholds for 2.36 and 2 fold change values. The green spots are the differentially expressed outlier genes for healthy *vs. *healthy with obesity comparison.

The distribution of log fold change values was obtained and the outliers were detected for the optimum cut-off value (c*). Figure 6 shows the thresholds for 2.36-fold change, thereby providing the up and down regulated genes. Out of 3,940 outlier genes, 1,247 were detected as up regulated, while 331 were detected as down-regulated genes with respect to the healthy (H) individual. Thus, for healthy vs. healthy with obesity comparison, 1,578 genes were found to be differentially expressed out of 39,400, which amounts to 4% of the total genes under study. This is 2.7% less than the number of genes obtained for 2-fold change thresholds. We refer 2.36 as the modified fold change, which is obtained based on the scatter of the distribution. More the scatter, larger is the modified fold change, thereby reducing the number of DE genes. The up and down regulated genes for the two categories were further considered for Gene Ontology (GO) and pathway analysis. On similar lines, the analysis was carried out for the remaining fourteen comparisons and the corresponding figures for each comparison are given below.

### 2. Healthy (reference) *vs *Obesity (test sample) [H *vs *O]

**Figure 7:**
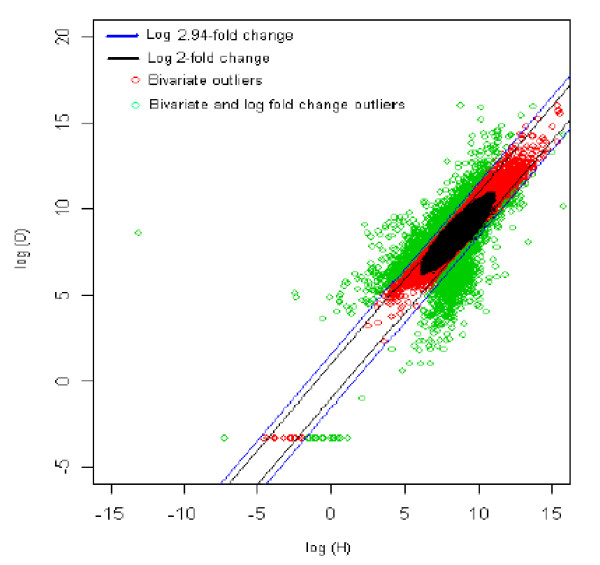
The thresholds for 2.94 and 2 fold change values. The green spots are the differentially expressed outlier genes for healthy *vs. *obesity comparison.

The distribution of log fold change values was obtained and the outliers were detected for the optimum cut-off (c*). Figure 7 shows the thresholds for 2.94-fold change, thereby providing the up and down regulated genes. Out of 3,940 outlier genes, 962 were detected as up regulated, while 989 were detected as down-regulated genes with respect to the healthy (H) individual. Thus, for healthy vs. obesity comparison, 1,951 genes were found to be differentially expressed out of 39,400, which amounts to 4.9% of the total genes under study. This is 6% less than the number of genes obtained for 2-fold change thresholds.

### 3. Healthy (reference) *vs *Diabetic with no parental history1 (test sample) [H *vs *DNPH1]

**Figure 8:**
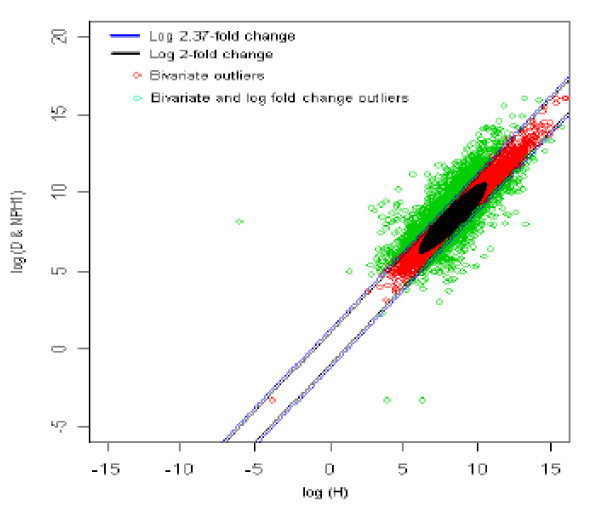
The thresholds for 2.37 and 2 fold change values. The green spots are the differentially expressed outlier genes for healthy *vs. *diabetic with no parental history [1] comparison.

The distribution of log fold change values was obtained and the outliers were detected for the optimum cut-off value (c*). Figure 8 shows the thresholds for 2.37-fold change, thereby providing the up and down regulated genes. Out of 3,940 outlier genes, 1,249 were detected as up regulated, while 477 were detected as down-regulated genes with respect the healthy (H) individual. Thus, for healthy vs. diabetic with no parental history (1) comparison, 1,726 genes were found to be differentially expressed out of 39,400, which amounts to 4.3% of the total genes under study. This is 3.3% less than the number of genes obtained for 2-fold change thresholds.

### 4. Healthy (reference) *vs *Diabetic with no parental history2 (test sample) [H *vs *DNPH2]

**Figure 9:**
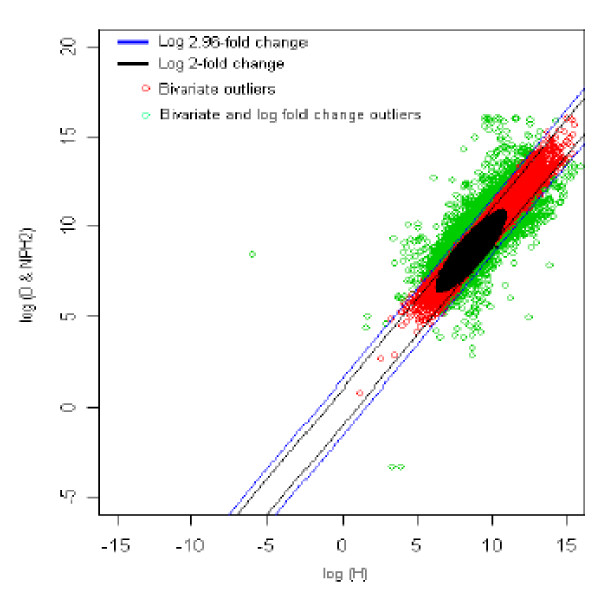
The thresholds for 2.96 and 2 fold change values. The green spots are the differentially expressed outlier genes for healthy vs. diabetic with no parental history [2] comparison.

The distribution of log fold change values was obtained and the outliers were detected for the optimum cut-off value (c*). Figure 9 shows the thresholds for 2.96-fold change, thereby providing the up and down regulated genes. Out of 3,940 outlier genes, 861 were detected as up regulated, while 356 were detected as down-regulated genes with respect to the healthy (H) individual. Thus, for healthy vs. diabetic with no parental history [2] comparison, 1,217 genes were found to be differentially expressed out of 39,400, which amounts to 3% of the total genes under study. This is 7% less than the number of genes obtained for 2-fold change thresholds. It can be seen from the results depicted in Figures 8 and 9 that even though both subjects [1 and 2] are having type 2 diabetes mellitus with no parental history of diabetes, the genes that were differentially expressed compared to healthy control were slightly different between the two [1 and 2]. But, on the whole the differentially expressed genes were similar (compare figure 8 and 9).

### 5. Healthy (reference) *vs *Diabetic with parental history (test sample) [H *vs *DPH]

**Figure 10:**
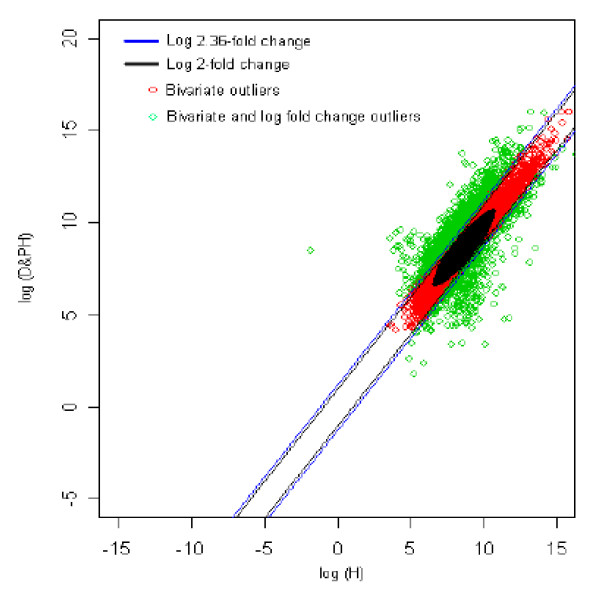
The thresholds for 2.36 and 2 fold change values. The green spots are the differentially expressed outlier genes for healthy vs. diabetic with parental history of type 2 diabetes mellitus comparison.

The distribution of log fold change values was obtained and the outliers were detected for the optimum cut-off value (c*). Figure 10 shows the thresholds for 2.36-fold change, thereby providing the up and down regulated genes. Out of 3,940 outlier genes, 1,211 were detected as up regulated, while 368 were detected as down-regulated genes with respect to the healthy (H) individual. Thus, for healthy vs. diabetic with parental history comparison, 1,579 genes were found to be differentially expressed out of 39,400, which amounts to 4% of the total genes under study. This is 2.73% less than the number of genes obtained for 2-fold change thresholds.

### 6. Healthy with overweight (reference) *vs *Obesity (test sample) [HO *vs *O]

**Figure 11:**
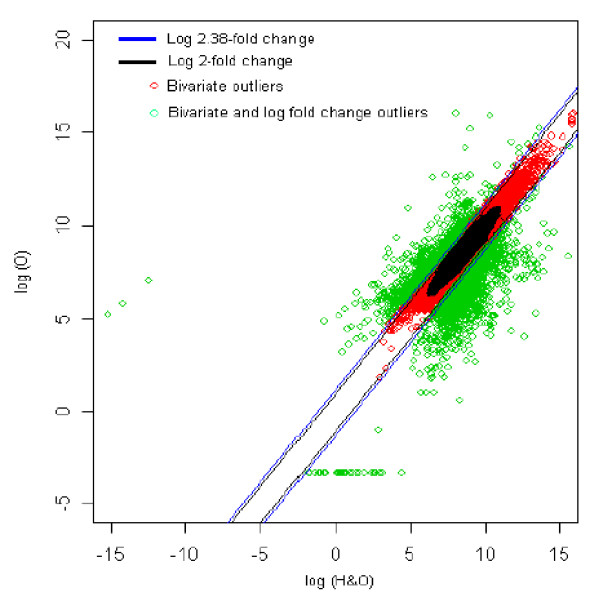
The thresholds for 2.38 and 2 fold change values. The green spots are the differentially expressed outlier genes for healthy with overweight s. obesity comparison.

The distribution of log fold change values was obtained and the outliers were detected for the optimum cut-off value (c*). Figure 11 shows the thresholds for 2.38-fold change, thereby providing the up and down regulated genes. Out of 3,940 outlier genes, 814 were detected as up regulated, while 1,469 were detected as down-regulated genes with respect to healthy individual with overweight (HO). Thus, for healthy with obesity vs. obesity comparison, 2,283 genes were found to be differentially expressed out of 39,400, which amounts to 5.8% of the total genes under study. This is 2.6% less than the number of genes obtained for 2-fold change thresholds.

### 7. Healthy with Overweight (reference) *vs *Diabetic with no parental history1 (test sample) [HO *vs *DNPH1]

**Figure 12:**
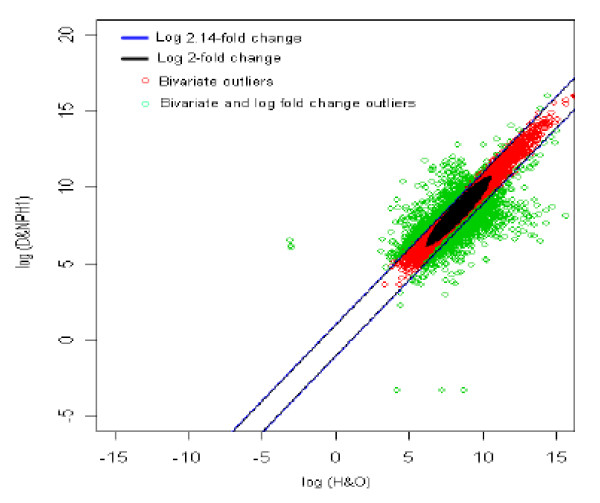
The thresholds for 2.14 and 2 fold change values. The green spots are the differentially expressed outlier genes for healthy with overweight vs. diabetic with no parental history [1] comparison.

The distribution of log fold change values was obtained and the outliers were detected for the optimum cut-off value (c*). Figure 12 shows the thresholds for 2.14-fold change, thereby providing the up and down regulated genes. Out of 3,940 outlier genes, 539 were detected as up regulated, while 1,058 were detected as down-regulated genes with respect to healthy individual with overweight (HO). Thus, for healthy with overweight *vs. *diabetic with no parental history [1] comparison, 1,597 genes were found to be differentially expressed out of 39,400, which amounts to 4% of the total genes under study. This is 1.2% less than the number of genes obtained for 2-fold change thresholds.

### 8. Healthy with Overweight (reference) *vs *Diabetic with no parental history2 (test sample) [HO *vs *DNPH2]

**Figure 13:**
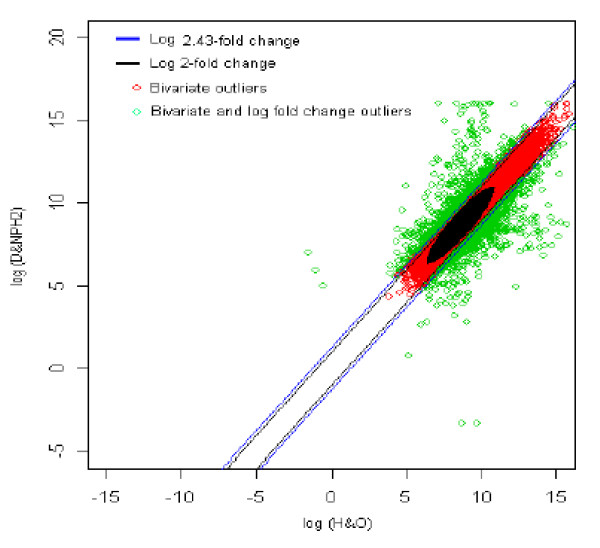
The thresholds for 2.43 and 2 fold change values. The green spots are the differentially expressed outlier genes for healthy with overweight vs. diabetic with no parental history [2] comparison. Compare these results with those in Figure 12.

The distribution of log fold change values was obtained and the outliers were detected for the optimum cut-off value (c*). Figure 13 shows the thresholds for 2.43-fold change, thereby providing the up and down regulated genes. Out of 3,940 outlier genes, 541 were detected as up regulated, while 672 were detected as down-regulated genes with respect to healthy individual with obesity (HO). Thus, for healthy with overweight *vs. *diabetic with no parental history [2] comparison, 1,213 genes were found to be differentially expressed out of 39,400, which amounts to 3% of the total genes under study. This is 2.75% less than the number of genes obtained for 2-fold change thresholds. When the results given in Figures 12 and 13 are compared they look very similar. This suggests that, in general, the differentially expressed genes between healthy with overweight vs. those with type 2 diabetes mellitus (even when compared with two distinctly different individuals) with no parental history are almost identical.

### 9. Healthy with Overweight (reference) *vs *Diabetic with parental history (test sample) [HO *vs *DPH]

**Figure 14:**
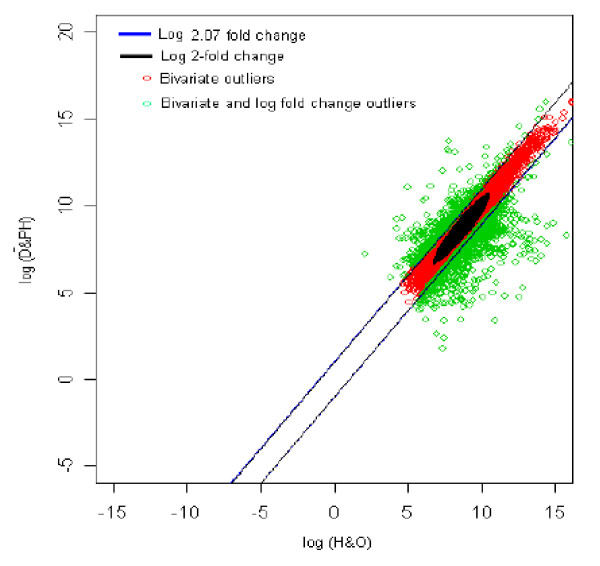
The thresholds for 2.07 and 2 fold change values. The green spots are the differentially expressed outlier genes for healthy with overweight vs. diabetic with parental history of diabetes comparison.

The distribution of log fold change values was obtained and the outliers were detected for the optimum cut-off value (c*). Figure 14 shows the thresholds for 2.07-fold change, thereby providing the up and down regulated genes. Out of 3,940 outlier genes, 502 were detected as up regulated, while 822 were detected as down-regulated genes with respect to healthy individual with overweight (HO). Thus, for healthy with overweight *vs. *diabetic with parental history comparison, 1,324 genes were found to be differentially expressed out of 39,400, which amounts to 3.3% of the total genes under study. This is 0.05% less than the number of genes obtained for 2-fold change thresholds.

### 10. Obesity (reference) *vs *Diabetic with no parental history1 (test sample) [O *vs *DNPH1]

**Figure 15:**
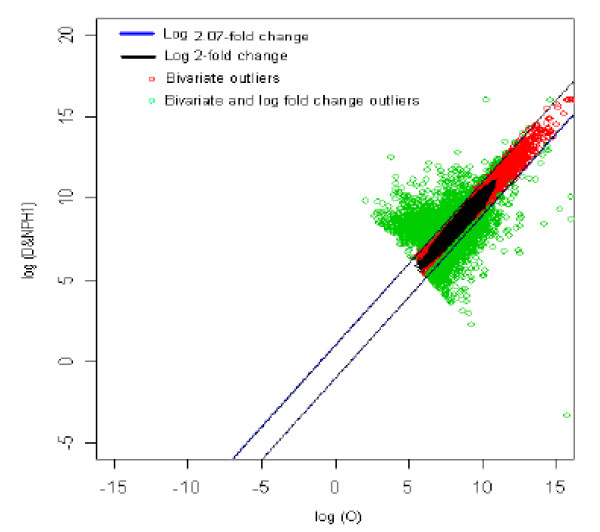
The thresholds for 2.07 and 2 fold change values. The green spots are the differentially expressed outlier genes for obesity vs. diabetic with no parental history [1] of diabetes comparison.

The distribution of log fold change values was obtained and the outliers were detected for the optimum cut-off (c*). Figure 15 shows the thresholds for 2.07-fold change, thereby providing the up and down regulated genes. Out of 3,940 outlier genes, 1,479 were detected as up regulated, while 1,333 were detected as down-regulated genes with respect the individual with obesity (O). Thus, for obesity *vs. *diabetes with no parental history [1] comparison, 2,812 genes were found to be differentially expressed out of 39,400, which amounts to 7.1% of the total genes under study. This is 0.06% less than the number of genes obtained for 2-fold change thresholds.

### 11. Obesity (reference) *vs *Diabetic with no parental history2 (test sample) [O *vs *DNPH2]

**Figure 16:**
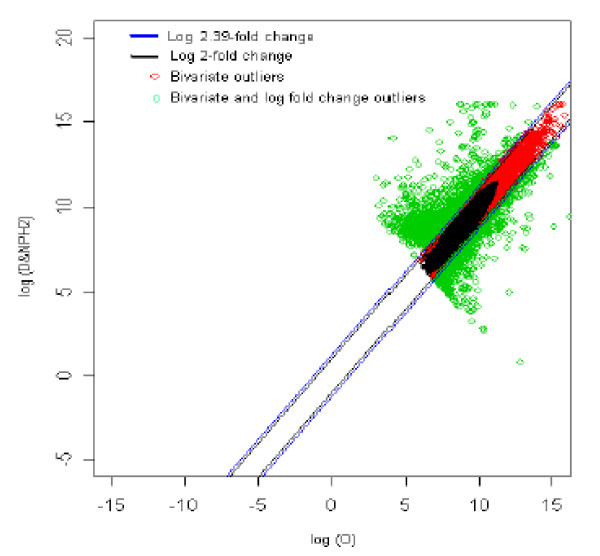
The thresholds for 2.39 and 2 fold change values. The green spots are the differentially expressed outlier genes for obesity vs. diabetic with no parental history [2] of diabetes comparison.

The distribution of log fold change values was obtained and the outliers were detected for the optimum cut-off value (c*). Figure 16 shows the thresholds for 2.39-fold change, thereby providing the up and down regulated genes. Out of 3,940 outlier genes, 1,534 were detected as up regulated, while 813 were detected as down-regulated genes with respect the individual with obesity (O). Thus, for obesity *vs. *diabetic with no parental history [2] comparison, 2,347 genes were found to be differentially expressed out of 39,400, which amounts to 5.95% of the total genes under study. This is 2.6% less than the number of genes obtained for 2-fold change thresholds.

### 12. Obesity (reference) *vs *Diabetic with parental history (test sample) [O *vs *DPH]

**Figure 17:**
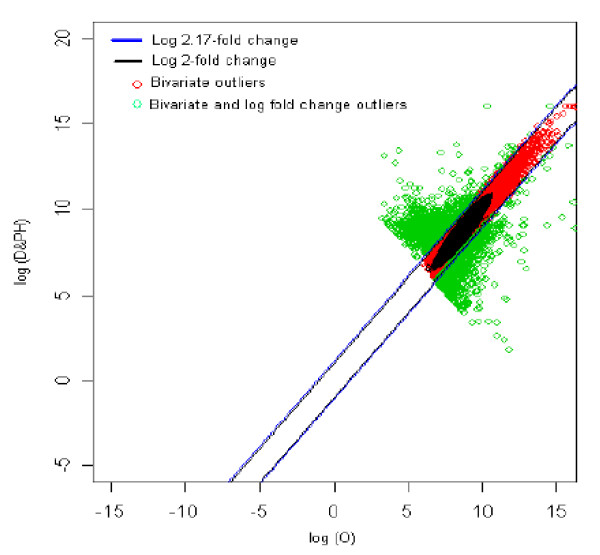
The thresholds for 2.17 and 2 fold change values. The green spots are the differentially expressed outlier genes for obesity vs. diabetic with no parental history comparison.

The distribution of log fold change values was obtained and the outliers were detected for the optimum cut-off value (c*). Figure 17 shows the thresholds for 2.17-fold change, thereby providing the up and down regulated genes. Out of 3,940 outlier genes, 1,338 were detected as up regulated, while 1,002 were detected as down-regulated genes with respect the individual with obesity. Thus, for obesity vs. diabetic with parental history comparison, 2,340 genes were found to be differentially expressed out of 39,400, which amounts to 5.93% of the total genes under study. This is 1% less than the number of genes obtained for 2-fold change thresholds.

### 13. Diabetic with no parental history1 (reference) *vs *Diabetic with no parental history2 (test sample) [DNPH1 *vs *DNPH2]

**Figure 18:**
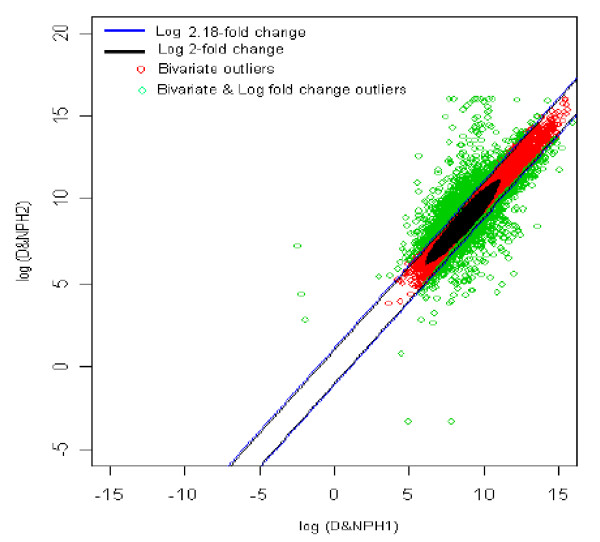
The thresholds for 2.18 and 2 fold change values. The green spots are the differentially expressed outlier genes when one diabetic with no parental history [1] was compared with another diabetic with no parental history [2].

Here we compared gene expression profile between two subjects with type 2 diabetes mellitus whose parents were also diabetic. The distribution of log fold change values was obtained and the outliers were detected for the optimum cut-off value (c*). Figure 18 shows the thresholds for 2.18-fold change, thereby providing the up and down regulated genes. Out of 3,940 outlier genes, 948 were detected as up regulated, while 662 were detected as down-regulated genes with respect to the individual with diabetes and no parental history [1]. Thus, a comparison between two individuals who were both diabetic with no parental history [1 and 2], 1,610 genes were found to be differentially expressed out of 39,400, which amounts to 4% of the total genes under study. This is 1.5% less than the number of genes obtained for 2-fold change thresholds.

### 14. Diabetic with no parental history1 (reference) *vs *Diabetic with parental history (test sample) [DNPH1 *vs *DPH]

**Figure 19:**
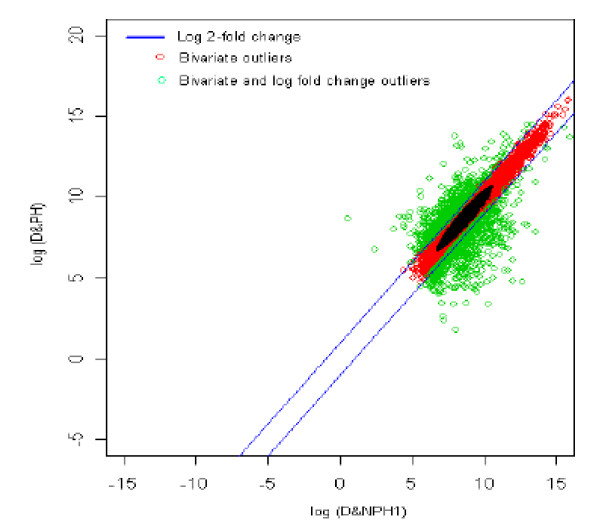
The thresholds for 2-fold change values. The green spots are the differentially expressed outlier genes for diabetic with no parental history [1] vs. diabetic with parental history comparison. Here the modified threshold was same as conventional 2-fold change.

The distribution of log fold change values was obtained and the outliers were detected for the optimum cut-off value (c*). Figure 19 shows the thresholds for 2-fold change, thereby providing the up and down regulated genes. Out of 3,940 outlier genes, 686 were detected as up regulated, while 682 were detected as down-regulated genes with respect to the individual with diabetic and no parental history [1]. Thus, for diabetic with no parental history [1] vs. diabetic with parental history comparison, 1,368 were found to be differentially expressed out of 39,400, which amounts to 3.4% of the total genes under study.

### 15. Diabetic with no parental history2 (reference) *vs *Diabetic with parental history (test sample) [DNPH2 *vs *DNPH]

**Figure 20:**
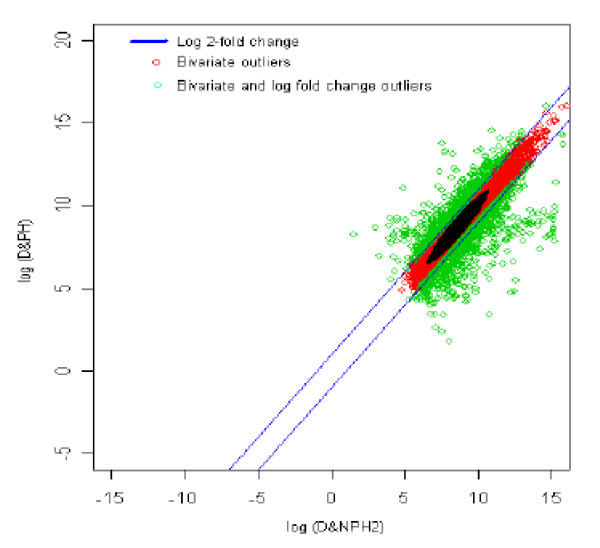
The thresholds for 2-fold change values. The green spots are the differentially expressed outlier genes for diabetic with no parental history [2] vs diabetic with parental history. Here the modified threshold was same as conventional 2-fold change.

The distribution of log fold change values was obtained and the outliers were detected for the optimum cut-off value (c*). Figure 20 shows the thresholds for 2-fold change, thereby providing the up and down regulated genes. Out of 3,940 outlier genes, 676 were detected as up regulated, while 979 were detected as down-regulated genes with respect to the individual with diabetic and no parental history [2]. Thus, for diabetic with no parental history [2] vs. diabetic with parental history comparison, 1,655 were found to be differentially expressed out of 39,400, which amounts to 4.2% of the total genes under study. When figures 19 and 20 are compared, it is clear that the differentially expressed genes between diabetics with no parental history [subjects 1 and 2] vs diabetic with parental history are very similar. This suggests that even though subjects with diabetes with no parental history are different the expressions of genes are closely similar when compared with those seen in diabetics with parental history (see Figures 19 and 20).

### Functional classification of differentially expressed genes

To determine biological significance of differentially expressed genes, functional classification was performed using Gene Ontology. Gene Ontology reports along with Z-scores give statistical significance, indicating relative representation up-regulated/down-regulated genes in each function. To determine pathways associated with differentially expressed genes, pathway analysis was performed.

### Gene Ontology Analysis

#### 1. Diabetes with parental history *vs *Normal [DPH *vs *H]

##### Molecular Function

Genes involved in NADH dehydrogenase (ubiquinone) activity, glutamate dehydrogenase [NAD(P)+] activity, CDP-diacylglycerol-glycerol-3-phosphate-3-phosphtidyltransferase activity were upregulated in DPH with respect to H. Genes involved in protein kinase B binding, enzyme inhibitor activity, acyl-CoA oxidase activity, phosphatidylinositol transporter activity, acyltransferase activity were downregulated in DPH with respect to H.

##### Biological Process

Genes involved in synaptic vesicle membrane organization and biogenesis, polysaccharide metabolic process, regulation of growth rate, nucleosome assembly were upregulated in DPH with respect to H. Genes involved in immune response, regulation of glycolysis were downregulated in DPH with respect to H.

##### Cellular Component

Genes localized in cohesin core heterodimer, oligosaccharyl transferase complex, nucleosome, respiratory chain complex II were upregulated in DPH with respect to H. Genes localized in isoamylase complex, protein kinase CK2 complex, proteasome activator complex, 6-phosphofructokinase complex were downregulated in DPH with respect to H.

#### 2. Diabetes without parental history1 *vs *Normal [DNPH1 *vs *H]

##### Molecular Function

Genes involved in hydroxyacylglutathione hydrolase activity, NADH dehydrogenase (ubiquinone) activity, GABA-B receptor activity, glutamate dehydrogenase [NAD(P)+] activity, CDP-diacylglycerol-glycerol-3-phosphate-3-phosphatidyl transferase activity were upregulated in DNPH1 with respect to H. Genes involved in MHC class II receptor activity, structural constituent of ribosome, Hsp70 protein binding, L-tyrosine transporter activity, cyclin binding, arachidonate 5-lipoxygenase activity were downregulated in DNPH1 with respect to H.

##### Biological Process

Genes involved in synaptic vesicle membrane organization and biogenesis, plasma membrane organization and biogenesis, polysaccharide metabolic process, regulation of growth rate, regulation of pH were upregulated in DNPH1 with respect to H. Genes involved in establishment of cellular localization, cell activation, immune response were downregulated in DNPH1 with respect to H.

##### Cellular Component

Genes localized in vacuolar lumen, chromosome, nucleosome, proteasome activator complex were upregulated in DNPH1 with respect to H. Genes localized in ferritin complex, proton-transporting ATP synthase complex, coupling factor F(o), ribosome, eukaryotic translation elongation factor 1 complex, ubiquitin conjugating enzyme complex were downregulated in DNPH1 with respect to H.

#### 3. Type 2 diabetes mellitus without parental history2 *vs *Normal [DNPH2 *vs *H]

##### Molecular Function

Genes involved in asparaginase activity, creatine: sodium symporter activity, phosphomannomutase activity, glutamate dehydrogenase [NAD(P)+] activity, basic amino acid transporter activity, adenylosuccinate synthase activity were upregulated in D&NPH2 with respect to H. Genes involved in structural constituent of ribosome, MHC class II receptor activity, MHC class I receptor activity, L-tyrosine transporter activity, N-acylmannosamine kinase activity were downregulated in D&NPH2 with respect to H.

##### Biological Process

Genes involved in polysaccharide metabolic process, regulation of pH, aromatic compound biosynthetic process, regulation of growth rate, lipid glycosylation were upregulated in D&NPH1 with respect to H. Genes involved in establishment of cellular localization, immune response, ribosome biogenesis and assembly were downregulated in D&NPH2 with respect to H.

##### Cellular Component

Genes localized in 4-aminobutyrate transaminase complex, oligosaccharyl transferase complex were upregulated in D&NPH1 with respect to H. Genes localized in ribosome, Arp2/3 protein complex, eukaryotic translation elongation factor 1 complex, small ribosomal subunit, ferritin complex, mitochondrial outer membrane translocase complex were downregulated in D&NPH2 with respect to H.

#### 4. Obese *vs *Normal (O *vs *H)

##### Molecular Function

Genes involved in peptide deformylase activity, NADH dehydrogenase (ubiquinone) activity, glutamate dehydrogenase [NAD(P)+] activity, phosphomannomutase activity, transposase activity, carboxylic ester hydrolase activity, glutamate decarboxylase activity, mannosyltransferase activity, transforming growth factor beta binding were upregulated in O with respect to H. Genes involved in glycolipid transporter activity, glycolipid binding, 3-hydroxyisobutyrate dehydrogenase activity, 25-hydroxycholecalciferol-24-hydroxylase activity were downregulated in O with respect to H.

##### Biological Process

Genes involved in regulation of isoprenoid metabolic process, polysaccharide metabolic process, regulation of pH were upregulated in O with respect to H. Genes involved in synaptic vesicle membrane organization and biogenesis, cellular macromolecule catabolic process, locomotion during locomotor behavior were downregulated in O with respect to H.

##### Cellular Component

Genes localized in CAAX-protein geranylgeranyltransferase complex, intracellular organelle were upregulated in O with respect to H. Genes localized in vesicle, eukaryotic translation elongation factor 1 complex, perikaryon, Golgi transport complex are downregulated in O with respect to H.

#### 5. Diabetes with parental history *vs *Obese [DPH *vs *O]

##### Molecular Function

Genes involved in glycolipid transporter activity, calmodulin inhibitor activity, glycolipid binding, interleukin-22 receptor activity, oxygen transporter activity, antigen binding, lactate dehydrogenase activity, glyoxylate reductase (NADP) activity, 25-hydroxycholecalciferol-24-hydroxylase activity, glycerate dehydrogenase activity, ubiquinol-cytochrome-c reductase activity were upregulated in DPH with respect to O. Genes involved in amylo-alpha-1, 6-glucosidase activity, 4-alpha-glucanotransferase activity, interleukin-8 receptor activity were downregulated in DPH with respect to O.

##### Biological Process

Genes involved in synaptic vesicle membrane organization and biogenesis, response to stimulus, cellular macromolecule catabolic process were upregulated in DPH with respect to O. Genes involved in regulation of isoprenoid metabolic process, blastocyst growth, regulation of glycolysis were downregulated in DPH with respect to O.

##### Cellular Component

Genes localized in vesicle hemoglobin complex, perikaryon, Golgi transport complex were upregulated in DPH with respect to O. Genes localized in isoamylase complex, CAAX-protein geranylgeranyltransferase complex, NADPH oxidase complex, protein kinase CK2 complex, MHC class I peptide loading complex, proteasome activator complex are downregulated in DPH with respect to O.

#### 6. Genes that were similarly expressed both in diabetes and obesity [DPH and O]

##### Molecular Function

Genes involved in NADH dehydrogenase (ubiquinone) activity, glutamate dehydrogenase [NAD(P)+] activity, transposase activity, guanylate cyclase inhibitor activity were upregulated both in diabetes and obesity. Genes involved in hypoxanthine phosphoribosyltransferase activity, structural constituent of ribosome, NADP binding, histone deacetylase activity were downregulated in diabetes and obesity.

##### Biological Process

Genes involved in polysaccharide metabolic process, regulation of pH, tissue development, and diuresis were upregulated both in diabetes and obesity. Genes involved in regulation of hormone biosynthetic process, opsonization were downregulated in diabetes and obesity.

##### Cellular Component

Genes localized in oligosaccharyl transferase complex, cytoplasmic vesicle, ribosome are upregulated in diabetes and obesity. Genes localized in small ribosomal subunit, proton-transporting ATP synthase complex, coupling factor F(o) were downregulated in diabetes and obesity.

#### 7. Obese *vs *overweight subjects with tendency towards Obesity (O *vs *HO)

##### Molecular Function

Genes involved in transforming growth factor beta binding, sodium: amino acid symporter activity, adenosylhomocysteinase activity, transferase activity, transferring acyl groups, caspase activator activity, NAD(P)H oxidase activity,

steroid 21-monooxygenase activity, malate dehydrogenase (oxaloacetate-decarboxy-lating) (NADP+) activity, glutamate decarboxylase activity were upregulated in O Vs HO. Genes involved in creatine: sodium symporter activity, glycolipid transporter activity, glycolipid binding, 3-hydroxyisobutyrate dehydrogenase activity, leukemia inhibitory factor receptor activity, superoxide-generating NADPH oxidase activity, chemokine receptor activity, interleukin-22 receptor activity were downregulated in O *vs *HO.

##### Biological Process

Genes involved in establishment of cellular localization, cuticle biosynthetic process, hydrogen peroxide, biosynthetic process, vesicle docking were upregulated in O *vs *HO. Genes involved in synaptic vesicle membrane organization and biogenesis, response to stimulus, anatomical structure development were downregulated in O *vs *HO.

##### Cellular Component

Genes localized in CAAX-protein geranylgeranyltransferase complex are upregulated in O vs HO. Genes localized in Golgi transport complex, vesicle, oncostatin-M receptor complex, perikaryon were downregulated in O vs HO.

#### 8. Diabetes with parental history *vs *diabetes without parental history (DPH *vs *DNPH1)

##### Molecular Function

Genes involved in MHC class II receptor activity, gamma-aminobutyric acid: hydrogen symporter activity, chemokine receptor activity, interleukin-4 receptor activity, interleukin-7 receptor activity, arachidonate 5-lipoxygenase activity, complement receptor activity were upregulated in DPH *vs *DNPH1. Genes involved in ammonia ligase activity, transaldolase activity, 4-alpha-glucanotransferase activity, choline: sodium symporter activity, interleukin-8 receptor activity were downregulated in DPH *vs *DNPH1.

##### Biological Process

Genes involved in cell activation, macromolecule biosynthetic process, hydrogen peroxide biosynthetic process, immune response, regulation of glycolysis were upregulated in DPH Vs DNPH1. Genes involved in blastocyst growth, aromatic compound biosynthetic process, nitric oxide biosynthetic process, regulation of glycolysis were downregulated in DPH *vs *DNPH1.

##### Cellular Component

Genes localized in ribonucleosidediphosphate reductase complex, interleukin-18 receptor complex, interleukin-1 receptor complex, mitochondrion interleukin-5 receptor complex were upregulated in DPH *vs *DNPH1. Genes localized in proteasome activator complex, isoamylase complex, CAAX-protein geranylgeranyl-transferase complex, protein kinase CK2 complex, oxoglutarate dehydrogenase complex, MHC class I peptide loading complex were downregulated in DPH *vs *DNPH1.

#### 9. Diabetic with parental history of diabetes *vs *diabetic with no parental history of diabetes (DPH *vs *DNPH2)

##### Molecular Function

Genes involved in structural constituent of ribosome, MHC class II receptor activity, ferroxidase activity, NAD(P)H oxidase activity were upregulated in DPH vs DNPH2. Genes involved in 4-alpha-glucanotransferase activity, phospho-mannomutase activity, receptor signaling protein tyrosine kinase activity were downregulated in DPH *vs *DNPH2.

##### Biological Process

Genes involved in intracellular sequestering of iron ion, ribosome biogenesis and assembly, hydrogen peroxide biosynthetic process were upregulated in DPH vs DNPH2. Genes involved in hemostasis, developmental growth, lipid glycosylation, regulation of glycolysis were downregulated in DPH *vs *DNPH2.

##### Cellular Component

Genes localized in ribosome, ferritin complex are upregulated in DPH vs DNPH2. Genes localized in CAAX-protein geranylgeranyltransferase complex, isoamylase complex, apolipoprotein B mRNA editing enzyme complex, lipopoly-saccharide receptor complex, proteasome activator complex are downregulated in DPH *vs *DNPH2.

For easy understanding, a summary of the gene ontology analysis data is given in Table 1.

**Table 1:** A summary of the gene ontology data with respect to molecular function is given here. Changes in the expression of genes concerned with biological processes and cellular components are given in the text.

S. No	Comparison	Up regulated genes	Down regulated genes
1	DPH vs H	NADH dehydrogenase (ubiquinone) Glutamate dehydrogenase [NAD(P)+] CDP-diacylglycerol-glycerol-3-phosphate-3-hosphitidltransferase	Protein kinase B binding Acyl-CoA oxidase Phosphatidylinositol transporter Acyltransferase
2	DNPH1 *vs *H	Hydroxyacylglutathione hydrolase NADH dehydrogenase (ubiquinone) GABA-B receptor Glutamate dehydrogenase [NAD(P)+] CDP-diacylglycerol-glycerol-3-phosphate-3-phosphatidyl transferase	MHC class II receptor Structural constituent of ribosome Hsp 70 protein binding L-tyrosine transporter Cyclin binding Arachidonate 5-lipoxygenase
3	DNPH2 *vs *H	Asparaginase activity Creatine: sodium symporter Phosphomannomutase activity Glutamate dehydrogenase [NAD(P)+] basic amino acid transporter Adenylosuccinate synthase	Structural constituent of ribosome MHC class II receptor MHC class I receptor L-tyrosine transporter N-acylmannosamine kinase
4	O *vs *H	Deformylase NADH dehydrogenase (ubiquinone) Glutamate dehydrogenase [NAD(P)+] Phosphomannomutase Transposase Carboxylic ester hydrolase Glutamate decarboxylase Mannosyltransferase Transforming growth factor beta binding	Glycolipid transporter activity Glycolipid binding 3-hydroxisobutyrate Dehydrogenase 25-hydroxycholecalciferol-24-hydroxylase
5	DPH *vs *O	Glycolipid transporter Calmodulin inhibitor Glycolipid binding Interleukin-22 receptor Oxygen transporter Antigen binding Lactate dehydrogenase Glyoxylate reductase (NADP) activity 25-hydroxycholecalciferol-24-hydroxylase activity Glycerate dehydrogenase Ubiquinol-cytochrome-c reductase	Amylo-alpha-1, 6 Glucosidase 4-alpha-glucanotransferase Interleukin-8 receptor activity
6	DPH and O	NADH dehydrogenase (ubiquinone) Glutamate dehydrogenase [NAD(P)+] Transposase Guanylate cyclase inhibitor	Hypoxanthine phosphoribosyltransferase Structural constituent of Ribosome NADP binding, and histone deacetylase
7	O *vs *HO	Transforming growth factor beta binding Sodium: amino acid symporter Adenosylhomocysteinase Transferase Transferring acyl groups Caspase activator NAD(P)H oxidase Steroid 21-monooxygenase Malate dehydrogenase (oxaloacetate-decarboxylating) (NADP+) Glutamate decarboxylase	Creatine: sodium symporter activity Glycolipid transporter Glycolipid binding, 3-hydroxyisobutyrate dehydrogenase Leukemia inhibitory factor receptor Superoxide-generating NADPH oxidase Chemokine receptor Interleukin-22 receptor
8	DPH *vs *DNPH1	MHC class II receptor Gamma-aminobutyric acid: hydrogen symporter chemokine receptor Interleukin-4 receptor Interleukin-7 receptor Arachidonate 5-lipoxygenase Complement receptor	Ammonia ligase Transaldolase 4-alpha-glucanotransferase Choline: sodium symporter Interleukin-8 receptor
9	DPH *vs *DNPH2	Structural constituent of ribosome MHC class II receptor Ferroxidase NAD(P)H oxidase	4-alpha-glucanotransferase Phospho-mannomutase Receptor signaling protein tyrosine kinase

#### Pathway Analysis

##### 1. Diabetes with parental history *vs *Normal [DPH *vs *H]

Genes involved in inositol phosphate metabolism, starch and sucrose metabolism, nitrogen metabolism, oxidative phosphorylation, androgen and estrogen metabolism, glycan biosynthesis and metabolism pathways, metabolism of cofactors and vitamins pathways, MAPK signaling pathway, ECM-receptor interaction, neuroactive ligand-receptor interaction, regulation of actin cytoskeleton, cell communication pathways, nervous system pathways, neurodegenerative disorders pathways were upregulated in DPH *vs *H. Genes involved in glycolysis/gluconeogenesis, propanoate metabolism, carbon fixation, biosynthesis of steroids, fatty acid metabolism, histidine metabolism, phenylalanine metabolism, tyrosine metabolism, urea cycle and metabolism of amino groups, cell cycle, insulin signaling pathway, PPAR signaling pathway, antigen processing and presentation were downregulated in DPH *vs *H.

##### Molecular Function

Genes involved in NADH dehydrogenase (ubiquinone) activity, glutamate dehydrogenase [NAD(P)+] activity, CDP-diacylglycerol-glycerol-3-phosphate-3-phosphtidyltransferase activity were upregulated in DPH with respect to H. Genes involved in protein kinase B binding, enzyme inhibitor activity, acyl-CoA oxidase activity, phosphatidylinositol transporter activity, acyltransferase activity were downregulated in DPH with respect to H.

#### 2. Diabetes without parental history *vs *Normal [DNPH1 *vs *H]

Genes involved in carbohydrate metabolism pathways, metabolism of cofactors and vitamins pathways, ubiquitin mediated proteolysis, signal transduction pathways, ECM-receptor interaction, neuroactive ligand receptor interaction, regulation of actin cytoskeleton, cell cycle, endocrine system pathways, nervous system pathways, Huntington's disease were upregulated in DNPH1 *vs *H. Genes involved in cell adhesion molecules (CAMs), antigen processing and presentation were downregulated in DNPH1 *vs *H.

#### 3. Diabetes without parental history *vs *Normal [DNPH2 *vs *H]

Genes involved in carbohydrate metabolism pathways, lipid metabolism pathways, glycan biosynthesis and metabolism pathways, metabolism of cofactors and vitamins pathways, ubiquitin mediated proteolysis, signal transduction pathways, signaling molecules and interaction pathways, PPAR signaling pathway, GnRH signaling pathway, nervous system pathways, development pathways, neurodegenerative disorders pathways were upregulated in DNPH2 *vs *H. Genes involved in insulin signaling pathway, immune system pathways were downregulated in DNPH2 *vs *H.

#### 4. Obese *vs *Normal [O *vs *H]

Genes involved in carbohydrate metabolism pathways, lipid metabolism pathways, amino acid metabolism pathways, glycan biosynthesis and metabolism pathways, metabolism of cofactors and vitamins pathways, ubiquitin mediated proteolysis, signal transduction pathways, neuroactive ligand-receptor interaction, nervous system pathways, neurodegenerative disorders pathways are upregulated in O *vs *H. Genes involved in cell adhesion molecules (CAMs), cytokine-cytokine receptor interaction, insulin signaling pathway, immune system pathways were downregulated in O *vs *H.

#### 5. Diabetes with parental history *vs *Obese [DPH *vs *O]

Genes involved in Inositol phosphate metabolism, oxidative phosphorylation, amino acid metabolism pathways, ubiquinone biosynthesis, signal transduction pathways, signaling molecules and interaction pathways, nervous system pathways were upregulated in DPH *vs *O.

#### 6. Diabetes with parental history *vs *diabetes without parental history (DPH *vs *DNPH1)

Genes involved in signal transduction, regulation of actin cytoskeleton, antigen processing and presentation, complement and coagulation cascades, axon guidance, Neurodegenerative disorders pathways were upregulated in DPH *vs *DNPH1.

Genes involved in carbohydrate pathways were downregulated in DPH vs DNPH1.

#### 7. Diabetes with parental history *vs *Diabetes with no parental history2 (DPH *vs *DNPH2)

Genes involved in oxidative phosphorylation, metabolism of cofactors and vitamins pathways, immune system pathways, nervous system pathways, metabolic disorders pathways were upregulated in DPH *vs *DNPH2. Genes involved in lipid metabolism pathways, amino acid metabolism pathways, glycan biosynthesis and metabolism pathways, ubiquitin mediated proteolysis, signal transduction pathways, signaling molecules and interaction pathways, insulin signaling pathway, PPAR signaling pathway were downregulated in DPH *vs *DNPH2.

### Changes in the expression of genes involved in inflammatory response

Plasma levels of CRP, TNF-α, and IL-6, which are markers of inflammation, are elevated in obesity, insulin resistance, essential hypertension, type 2 diabetes, and CHD both before and after the onset of these diseases [3-9]. In view of this, we specifically looked at the genes that are involved in inflammatory response in the present study. The data given in Table 2 depicts the inflammatory genes that were differentially expressed in obesity and type 2 diabetes mellitus.

**Table 2:** Genes involved in inflammatory response that were differentially expressed in subjects with obesity and type 2 diabetes mellitus.

**Condition**	**Differentially expressed genes concerned with inflammation**
Diabetic with family history *vs *Healthy individual (DPH *vs *H)	ALK, GCH1, IFIH1, IFIT1, IL11RA, ITGB2,MAP3K4, MMP19, MMP3, RPS27A, SLK, TNFRSF12A, UBC
Diabetic without family history *vs *healthy individual (DNPH1 *vs *H)	CCL3, CDKN1A, CXCL12, HLA-A, IL11RA, KRT8, LTB, MAP3K4, MMP10, MMP19, MMP20, MMP3, RPS27A, TNFSF10, UBC
Diabetic without family history Vs healthy individual (DNPH2 *vs *H)	CCL16, CCR8, CXCL11, CXCL12, FN1, GCH1, HLA-A, IL11RA, LTB, MMP19, MMP3, RHOA, S100A12, SLK, SYK, UBC
Obese vs Healthy (O *vs *H)	CCL13, CXCL12, HLA-A, IL6, KRT8, MMP19, MMP27, MMP3, RPS27A, UBC
Diabetic with family history *vs *obesity (DPH *vs *O)	ALK, CCL13, CCR4, CCR8, HGF, HLA-A, IFIT1, IL20, IL6, IL8RA, ITGB2, KRT8, MC1R, MMP20, MMP27, SLK, TNFRSF12A, UBC
Obese individual vs individual who is overweight (O *vs *HO)	CCL13, CCL16, CCR8, HLA-A, IFIT1, IL6, KRT8, MC1R, MMP16, MMP27, TNFRSF12A, UBC
Diabetic with family history *vs *Diabetic without family history (DPH *vs *DNPH1)	ALK, CCL13, CCR8, CDKN1A, EDN1, FGF1, IFIT1, IL12RB1, IL20, IL22, IL2RG, IL8RA, ITGB2, MMP20, SLK, TNFRSF12A, UBC, XCR1
Diabetic with family history *vs *Diabetic without family History2 (DPH *vs *DNPH2)	ALK, BLR1, CCL15, CCL16, CCR7, CCR8, CXCL11, CXCL12, FN1, FTH1, GBP1, HLA-A, IFIT1, IL12A, ITGB2, KIT, LTB, MMP20, PPARD, RHOA, RPS27A, TAC1, TLR4, TNFAIP6, TNFRSF11A

## Discussion

The incidence of obesity and type 2 diabetes mellitus and with it the consequent metabolic syndrome X is increasing throughout the world. It is estimated that by the year 2010, in the United States alone there may be about 50 to 75 million or more people with metabolic syndrome X. One important feature of metabolic syndrome X is the presence of insulin resistance. Subjects with abdominal obesity, hypertension, type 2 diabetes, hyperlipidemias, CHD, and stroke show insulin resistance and impaired glucose tolerance (IGT). Plasma levels of inflammatory markers such as CRP, TNF-α, and IL-6 are elevated in obesity, insulin resistance, essential hypertension, type 2 diabetes, CHD, and metabolic syndrome X [3-9], suggesting that all these disorders are associated with low-grade systemic inflammation. This is supported by the results of the present study wherein it was noted that genes involved in inflammatory response are differentially expressed in subjects with obesity and type 2 diabetes mellitus (see Table 2).

Early stages of obesity, type 2 diabetes mellitus, hypertension, and metabolic syndrome X are characterized by insulin resistance restricted to muscle tissue [16]. This may be the reason why exercise is beneficial in the prevention and treatment of insulin resistance since, it decreases insulin resistance and enhances glucose utilization in the muscles. Furthermore, exercise is anti-inflammatory in nature [17,18]. Exercise not only decreases the levels of inflammatory markers such as CRP, IL-6, and TNF-α but also simultaneously enhances the concentrations of anti-inflammatory cytokines IL-4, IL-10 and TGF-β compared to controls. IL-4, IL-10 and TGF-β are not only anti-inflammatory in nature but also suppress the production of pro-inflammatory cytokines IL-1, IL-2, and TNF-α [4].

Thus, under normal conditions there is a balance maintained between pro- and anti-inflammatory cytokines. In addition, in experimental animals exercise significantly reduced the magnitude of myocardial infarction and this cardioprotective action paralleled the increase in manganese superoxide dismutase (Mn-SOD) activity [19]. On the other hand, administration of antisense oligo-deoxyribonucleotide to Mn-SOD abolished this cardioprotective action implying that enhancement of the activity of Mn-SOD is crucial to exercise-induced cardioprotective action. This increase in Mn-SOD activity is in response to exercise-induced free radical generation suggesting that under certain circumstances free radicals have highly beneficial actions, especially when they are produced in response to exercise. Pro-inflammatory cytokines enhance free radical generation. It was noted that administration of antibodies to TNF-α and IL-1 abolished the cardioprotective action of exercise and activation of Mn-SOD, indicating that exercise-induced increase in the production of pro-inflammatory cytokines augment generation of free radicals that, in turn, enhance Mn-SOD activity that is ultimately responsible for the cardioprotective action of exercise. This is supported by the observation that circulating levels of extracellular SOD are lower in subjects with CHD [20]. These results are supported by the present observation that in patients with type 2 diabetes mellitus genes concerned with reactive oxygen species and pro-inflammatory cytokines such as BCL2L1, MAPK1, IL8RA, and IL-6 were up regulated whereas SOD2 was downregulated (see Table 3). In addition, it was also noted that TGFBR1 (transforming growth factor-β receptor 1), which is an anti-inflammatory cytokine that inhibits the production of pro-inflammatory cytokines such as IL-6 and TNF-α was found to be up regulated in type 2 diabetes, most probably as a compensatory mechanism. These results suggest that low-grade systemic inflammation plays a significant role in the pathobiology of obesity, type 2 diabetes mellitus, and metabolic syndrome X [1-9]. This is supported by the observation that weight loss achieved by type 2 diabetes subjects was associated not only with a decrease in glycosylated hemoglobin (HbA_1c_), LDL cholesterol, insulin resistance, plasminogen activator inhibitor-1, CRP, IL-6, and TNF-α but also with significant improvements in arterial stiffness [21] suggesting that endothelial nitric oxide (eNO) production is increased whereas oxidative stress is decreased. Thus, the results of the present study and other investigations indicate the genes concerned with inflammation and immune response are differentially regulated in subjects with obesity and type 2 diabetes mellitus.

**Table 3:** Pattern of gene expression observed in patients with type 2 diabetes mellitus with parental history (DPH) of diabetes *vs *healthy normal (H), and obese *vs *healthy.

Genes that have been down regulated in type 2 diabetes mellitus are:
MBL2, AKT1, ATP1A1, SOD2
Genes that have been up regulated in type 2 diabetes mellitus are:
ANXA1, NFKB2, GLUD1, BCL2L1, MAPK1, SOCS3, IRS2
Genes that have been up regulated in obese (O) compared to healthy (H) are:
IL8RA, TGFBR1, LITAF, IL-6, VEGFA

In this context, it is important to note that acetylcholine (ACh), the principal vagus neurotransmitter and an important neurotransmitter in the brain, is mostly concentrated in the brain, the spinal cord, and the rest of the nerve cells in the body and on the muscles of the body. The acetylcholine receptor modulates interactions between the nervous system and the immune system. Furthermore, the nervous system communicates with the immune system in a bi-directional pathway. Nervous tissues synthesize neuropeptides and cytokines and immune cells serve as the molecular basis of neural-immune interactions. Neural modulation can have both pro- and anti-inflammatory effects. ACh is known to have anti-inflammatory actions and suppress the production of pro-inflammatory cytokines [22] The cholinergic anti-inflammatory pathway signals through the efferent vagus nerve and mediates its actions primarily by nicotinic acetylcholine receptors on tissue macrophages leading to decreased NF-κB activation, preservation of high mobility group box 1 (HMGB1) nuclear localization and decreased production of proinflammatory cytokines. In addition, ACh has a regulatory role on serotonin, dopamine and other neuropeptides [23,24], suggesting that a complex network of interaction exists between these molecules in the regulation of immune response and neurotransmission.

Since under normal physiological conditions sympathetic and parasympathetic pathways demonstrate cross talk, it is pertinent to note that phagocytes are capable of *de novo *synthesis of catecholamines and blockade of α_2_-adrenoreceptors or catecholamine-generating enzymes suppressed inflammation [25]. Thus, parasympathetic nervous system suppresses inflammation by generating the anti-inflammatory molecule ACh, whereas sympathetic nervous system enhances the inflammatory response by secreting catecholamines. These results coupled with the observation that serotonin, another neurotransmitter, that has effects on behavior, mood, sleep, and appetite, has a direct role in the development and treatment of type 2 diabetes is interesting [26,27]. For instance, mice lacking the 5-HT2C receptor develop insulin resistance and type 2 diabetes and later overeat and become obese, whereas a drug that acts on 5-HT2C receptors improved glucose tolerance without leading to reductions in food intake or body weight by stimulating melanocyte-stimulating hormone (α-MSH) in the brain's arcuate nucleus, a portion of the hypothalamus that has a role in appetite control. These evidences are supported by the results of the present study wherein it was noted that genes involved in neuroactive ligand-receptor interaction, nervous system and neurodegenerative disorders pathways, axon guidance and immune and inflammatory pathways are altered.

In recent times since diet control, exercise, and drugs to reduce obesity are largely unsuccessful, the Roux-en-gastric bypass (RYGB) and other bariatric operations are becoming one of the most common abdominal surgical procedures in the USA [28]. RYGB produces on an average 49% to 65% weight loss within 2 to 5 years [29]. Besides weight loss, RYGB ameliorates diabetes, hyperlipidemia, and other obesity-related metabolic abnormalities [30,31]. While working with a rat model of RYGB in diet-induced obese rats, we observed that gastric bypass surgery produces significant weight loss due to reduced caloric intake with a reduction in meal size and meal number, accompanied by a decrease in serum glucose, insulin, leptin, triglyceride concentrations, and subcutaneous abdominal fat compared to the obese [32].

Further studies revealed that weight loss achieved by RYGB is in part due to a decrease in NPY (neuropeptide Y) in ARC (arcuate nucleus f hypothalamus), pPVN (parvocellular part of paraventricular nucleus of hypothalamus), and mPVN (magnocellular part of PVN) and an increase in α-MSH in ARC, pPVN, and mPVN compared with obese controls. 5HT-_1B_-receptor in pPVN and mPVN increased in RYGB and PF (pair fed) compared to obese control [33]. These results emphasize the fact that weight loss seen after RYGB and diet control is due to specific changes in hypothalamic peptides. Serotonin innervation is widely distributed in the hypothalamus and it innervates NPY neurons both in the ARC and PVN. Serotonin has a suppressive effect on food intake. Thus, weight loss seen in RYGB and diet control groups could be attributed to alterations in the concentrations of specific hypothalamic signaling peptides that regulate appetite, food intake and satiety. Even in tumor bearing anorectic rats, which showed significant weight loss due to tumor burden, similar results were seen: an increase of serotonin in PVN and VMN (ventromedial nucleus of hypothalamus) and a concomitant decrease of dopamine in PVN, VMN and LHA (lateral hypothalamus), and of NPY in LHA, VMN and PVN; a decrease in NPY in ARC and of POMC (proopiomelanocortin) in ARC and PVN [33,34] and these abnormalities reverted to normal after tumor resection. In this context, it is noteworthy that even the concentrations of IL-6 and TNF-α were found to be elevated in ARC in tumor-bearing rats. These results emphasize the close interaction(s) between neurotransmitters, inflammatory molecules, and obesity and type 2 diabetes mellitus [35].

Obviously the present results need to be verified using a larger sample size, by estimating the concentrations of the specific proteins of the genes expressed, and studying more closely the interaction(s) between the nervous system, hypothalamic peptides and neurotransmitters, pro- and anti-inflammatory cytokines, and their relationship to appetite, satiety, development of obesity and type 2 diabetes mellitus. It is also likely that there could be individual variations in the expression of various genes concerned with appetite, satiety, and inflammation and this need to be taken into consideration while assigning importance to the degree of expression of some of these genes. Such variations in the expression of genes could be detected only by performing studies in a larger sample size. Nevertheless, the results of the present study suggest that there are significant differences in the expression of various genes concerned with carbohydrate, lipid, and protein metabolism, ubiquitin mediated proteolysis, signal transduction pathways, neuroactive ligand-receptor interaction, nervous system pathways, cell adhesion molecules, cytokine-cytokine receptor interaction, insulin signaling and immune system pathways, oxidative phosphorylation, and PPAR signaling pathways in subjects with obesity and type 2 diabetes compared to normal.

## Authors' contributions

UND designed the study, analyzed the data, and drafted the manuscript. AAR performed the study, and drafted the manuscript. All authors read and approved the final manuscript.
